# Harnessing AI for aphasia: a case report on ChatGPT's role in supporting written expression

**DOI:** 10.3389/fresc.2025.1600145

**Published:** 2025-05-30

**Authors:** Avery K. Allen, Christine Brennan, Christina Riseman, Holly Kleiber, Allison I. Hilger

**Affiliations:** Department of Speech, Language, and Hearing Sciences, University of Colorado Boulder, Boulder, CO, United States

**Keywords:** aphasia, artificial intelligence, assistive technology, ChatGPT, language learning model, written language rehabilitation

## Abstract

**Introduction:**

Aphasia is associated with impairments in written language, including difficulty with sentence formulation, word finding, and editing. While writing aids show promise, artificial intelligence (AI) tools, such as large language models (LLMs), offer new opportunities for individuals with language-based writing challenges.

**Methods:**

This case report describes the use of the LLM, ChatGPT, to improve accuracy, complexity, and productivity in an adult with aphasia. The intervention combined self-genrated content with AI-assisted editing, guided by a visual flow char and structured prompts. Writing samples were analyzed for sentence count, complexity, and errors, while the patient's attitudes toward writing were evaluated through surveys.

**Results:**

When using ChatGPT, the patient produced more sentences with fewer errors, while self-written samples showed reduced total errors but decreased sentence production and increased sentence length and syntactic complexity. Although the patient required clinician prompting and modeling to use ChatGPT effectively, he developed greater independence and confidence over time. One year later, he reported continued use of ChatGPT for creative and communicative tasks.

**Discussion:**

This case highlights how AI tools can enhance written communication and promote participation in meaningful activities for individuals with aphasia, especially those with prior experience using technology.

## 1 Introduction

Aphasia is an acquired language disorder that arises from damage to the brain's language network. The hodotopical (delocalized) and dynamic model of language processing emphasizes the integration of cortical regions and white matter tracts as fundamental to language function (1). Accumulating evidence, including meta-analytic findings, links damage to various white matter pathways with impairments across multiple linguistic domains in people with aphasia (PWA) (2). Aphasia affects approximately two million people in the United States (3) and is associated with impairments in speech, comprehension, reading, and writing (4), contributing to challenges in mental health, social participation, healthcare access, and quality of life (5). While spoken language interventions have been widely researched, writing interventions are less explored and focus on the final product rather than the writing and editing process (6). There remains a gap in identifying effective approaches for addressing written expression. As artificial intelligence (AI) tools continue to grow, their potential to enhance written expression in this population warrants further investigation. This case report explores the use of the large language model (LLM), ChatGPT, to improve both the quantity and quality of written output in an adult with fluent aphasia.

Computer-based writing aids can support editing and word-finding in PWA (6). As AI tools, including LLMs, become more prevalent, speech-language pathologists (SLPs) and public health professionals are likely to encounter these tools more often in their work (7). Clinicians and medical practitioners use LLMs for differential diagnosis (8, 9), prognosis (10), and report writing and treatment planning (11). Using LLMs, such as ChatGPT, as a direct component of aphasia intervention is a relatively new area of study. PWA who used ChatGPT experienced improvements in word retrieval (7, 12). To date, no studies have investigated the use of an LLM specifically to enhance written output in PWA.

Writing is considered less automatic than speaking, making it more vulnerable to deficits following damage to the language network (13). Writing involves both low- and high-level processes: low-level processes include spelling, typing, and lexical retrieval, while high-level processes involve idea generation, evaluation, and revision (14). When low-level processes are automatized, more cognitive resources are available for high-level composition. When low-level writing processes are impaired due to aphasia, high-level composition is also affected (14, 15).

Though writing is impaired in aphasia, few studies have focused on this modality of language. Studies that do consider writing focus on abilities at the word level rather than at the sentence or narrative level (15). Compared to those without aphasia, PWA omitted critical information (16), had more syntactic errors (15), produced shorter texts (15), and had difficulties editing (15, 16).

There is a clear difference in narrative writing abilities between people with and without aphasia. Computer-based writing aids, such as those using spell-check and grammar, have positively impacted writing skills in PWA, particularly in word generation and revision abilities (17). The emergence of large language models (LLMs) presents new opportunities, building on the benefits of earlier computer-based aids. LLMs can generate human-like text and engage in conversational exchanges (18). In aphasia treatment, ChatGPT was found to assist with word identification and reduce circumlocution (7). A study exploring ChatGPT's potential in language disorders found it could support literacy and facilitate discussions on various topics (19).

This case study is informed by the International Classification of Functioning, Disability and Health (ICF) framework (20), which emphasizes a holistic view of health and disability by considering the disorder as well as impact on activity and participation, and environmental and personal factors. In the context of acquired cognitive-communication challenges, writing impairments are not solely a matter of linguistic deficits, but also reflect how individuals engage in meaningful communication activities and participate in everyday life. By examining the use of AI tools such as ChatGPT through the lens of the ICF, this study explores how such technologies may support not only specific language tasks, but also broader goals related to autonomy, self-expression, and social participation. Specifically, this case report addresses a gap in the literature on the use of LLMs to support written expression in PWA. Specifically, it evaluates the effectiveness of an AI-based writing intervention using ChatGPT for an adult with fluent aphasia, aiming to improve accuracy, complexity, and productivity in written output. The report details changes in written output and the patient's perception of the benefits of using ChatGPT as a writing tool.

## 2 Case description

**Figure 1:**
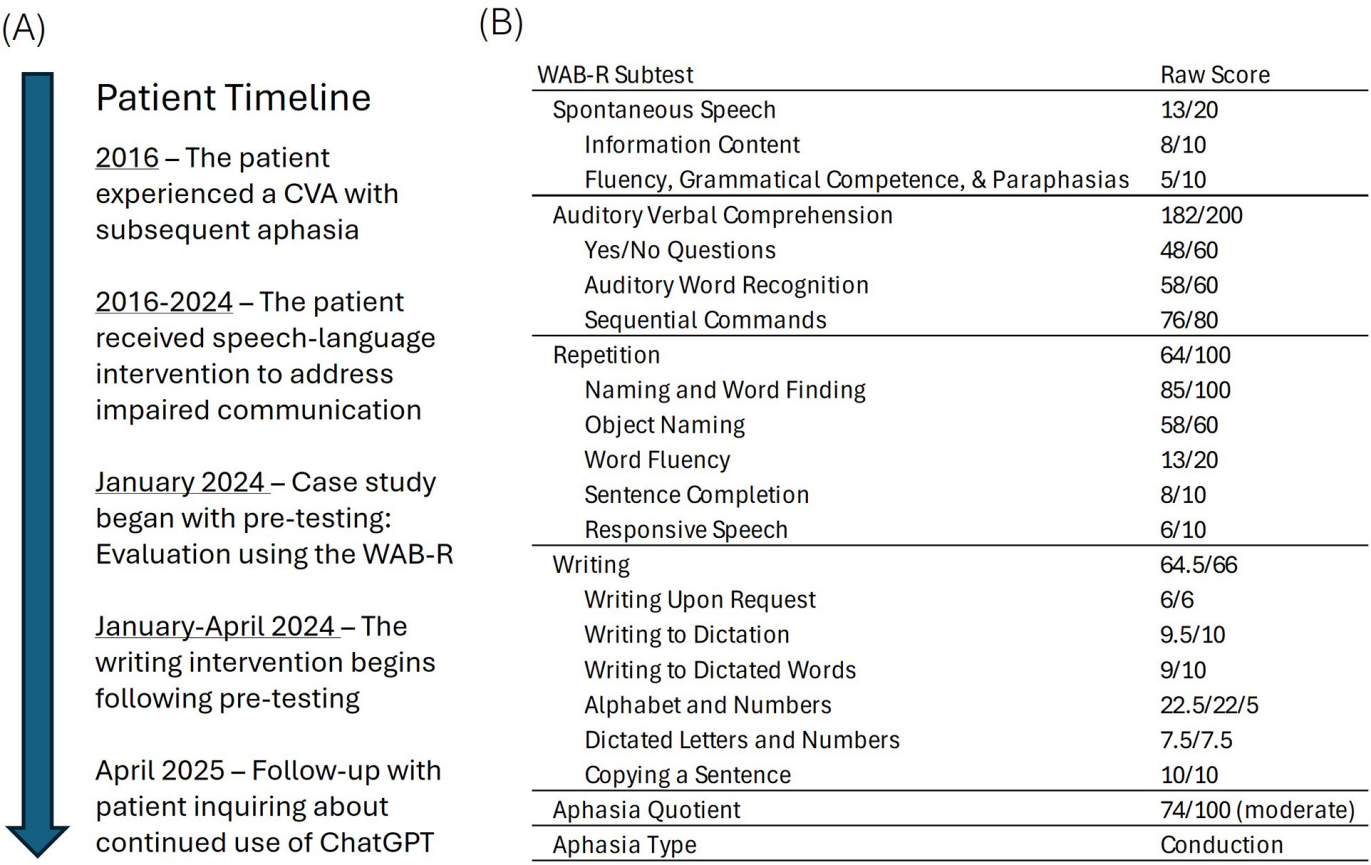
Case details including **(A)** patient timeline and **(B)** pre-testing assessment results.

The patient was a 75-year-old monolingual, English-speaking man with a previous clinical diagnosis of aphasia who experienced a left middle cerebral artery infarction eight years before participating in this study. He presented with difficulties in word finding and auditory comprehension and had bilateral hearing loss that required the use of hearing aids. He received speech-language therapy through a private speech-language clinic and a university clinic for eight years before this study (see Figure 1A).

The patient was separated/divorced and lived independently. He was able to drive himself to all appointments and managed appointments and finances without assistance. Socially, he played in a band and reported that he enjoyed eating out and listening to music. He kept in touch with friends via email and social media. At the time of the study, his participation in the aphasia support group was an important part of his social life post-stroke.

Before this study, written language treatment was introduced at the patient's request because he wanted to write his autobiography and was unable to formulate the narrative independently. The writing intervention using ChatGPT was offered to the patient as a way to help him accomplish his personal writing goals. Because of his prior experience with technology, he was eager to pursue this approach.

At the university clinic, the patient received individual and group therapy, provided by a graduate student clinician and supervised by a licensed SLP. Primary goals focused on improving auditory comprehension, expressive language, reading, conversation initiation, topic maintenance, and communicative participation strategies. The goal of the writing intervention was to have the patient learn to use ChatGPT effectively when writing and to increase his confidence and independence for writing. Additional aims included improving written language intelligibility and productivity. The clinician modeled the use of different ChatGPT prompts to increase the patient's independent use of this tool.

### 2.1 Intervention goals

Long-term goal 1: Mr. X will improve his ability to write and edit independently while maintaining accuracy.

Short-term goal 1.1: By the end of the semester, Mr. X will accurately state a topic sentence in 40% (2 of 5 opportunities) with moderate clinician verbal cueing.

Short-term goal 1.2: By the end of the semester, Mr. X will use the flow chart to accurately edit text in 3 of 5 opportunities with maximal clinician cueing.

Long-term goal 2: Mr. X will increase his confidence in writing.

Short-term goal 2.1: Mr. X will demonstrate an increase in his confidence in his writing ability as indicated by an average rating of 5 across the semester on the Likert scale below.

Question: How confident do you feel about your writing abilities?

Rating Scale:

1 = I Am not confident in my writing abilities.
2 = I Am minimally confident in my writing abilities.
3 = I Am somewhat confident in my writing abilities.
4 = I Am moderately confident in my writing abilities.
5 = I Am very confident in my writing abilities.
6 = I Am exceptionally confident in my writing abilities.
7 = I Am completely confident in my writing abilities.

### 2.2 Patient’s professional background

The patient's previous professional experiences included working as an educational designer and later as a software engineer who used early computer operating systems (i.e., MS-DOS). His work included design of user experience/user interfaces, development of computer conferencing software, and assisting businesses in designing their e-commerce platforms. Prior to this study, the patient was already using ChatGPT to compare and define topics while writing (specifically, by entering single words or phrases similar to how a simple search would be conducted using a search engine). He also used the speech-to-text feature on his smartphone to help with sentence generation and the writing program, Grammarly, for editing and spell-check.

### 2.3 Ethical consideration

The Institutional Review Board at BLINDED waives review of single-patient studies when intervention is provided as part of the patient's care. Informed verbal and written consent were obtained from the patient, and to protect the patient's privacy, no identifiable information is presented in this paper.

### 2.4 Assessment

A review of formal and informal evaluations, treatment notes, and progress on goals was conducted before the start began. Pre-testing results from the Western Aphasia Battery-Revised (WAB-R) (21) classified this patient with moderate conduction aphasia characterized by deficits in speech production, auditory comprehension, repetition, naming, and word finding (see Figure 1B). Language formulation challenges included difficulty with word finding and use of accurate syntax in the formulation of sentences (in the spoken and written modalities).

### 2.5 Intervention materials

**Figure 2:**
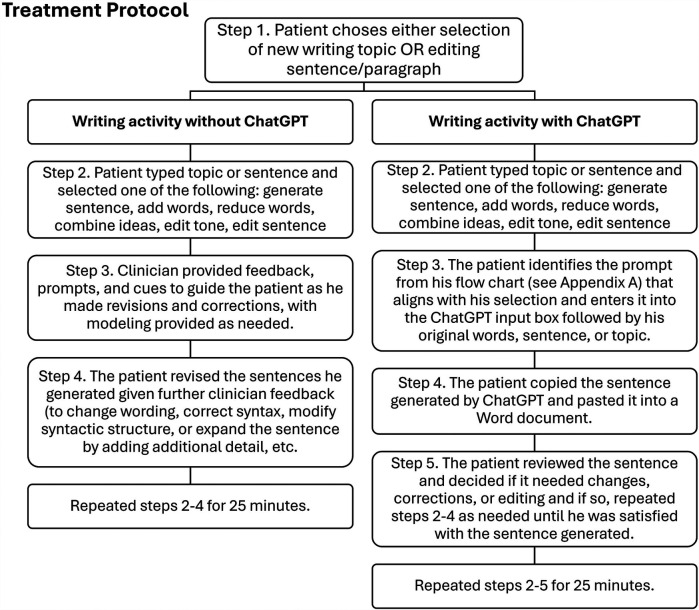
Treatment protocol for writing activities with and without ChatGPT.

A flow chart of prompts was created (see Supplementary Appendix A) for the patient to guide his writing process during AI writing activities. Clinician-led editing procedures (see Figure 2) helped the patient use these prompts for editing, synthesizing, expanding, contracting, correcting tone, creating headers, summarizing, and streamlining text.

Additional materials included a laptop, lined notebook, pencil, whiteboard, dry-erase marker, blank online document, and video-conferencing software. All sessions were video recorded, and the patient had access to the free version of ChatGPT-4 during each session.

### 2.6 Intervention procedures

The patient completed ten weekly 60 min sessions over 14 weeks at a university clinic, alongside participation in an aphasia therapy group focused on social communication and weekly individual speech-language therapy at a private practice. The only intervention targeting writing at the time of the study was provided at the university clinic, where a graduate student clinician, supervised by a licensed SLP, led the sessions.

Sessions started with selecting a writing topic. The patient used a laptop to create a new document each session. Each session included two intervention conditions, with and without ChatGPT. In the first 25 min, the patient generated sentences about the topic, either independently or with clinician assistance using the procedures outlined in Figure 2. In the next 25 min, writing included use of ChatGPT to edit, expand, or revise sentences (see Figure 2). The patient used the flow chart to select prompts for ChatGPT that aligned with his writing aims (see Supplementary Appendix A).

The final ten minutes of each session were dedicated to a survey assessing the patient's confidence, attitude, and mood regarding ChatGPT use (see Supplementary Appendix B). Modeled after the Scale for Mood Assessment (22), the survey included multiple-choice, Likert scale, and open-ended questions to evaluate changes in the patient's attitude, confidence, or mood following the intervention.

Clinician-led strategies included use of visual cues, graphic organizers, offering yes/no options, and using a whiteboard to write and illustrate word meanings and complex ideas.

### 2.7 Data collection

Two writing samples were collected from each session (one per condition). The patient completed the confidence and attitude survey at the end of each session. Writing samples were coded so that the following could be counted: simple sentences, complex sentences, the mean number of words per sentence, errors (including grammatical errors, wording errors, verb errors, pronoun errors, punctuation errors, compound sentence errors), and awkward wording (defined as phrasing that is unnatural, unclear, or difficult to understand).

### 2.8 Treatment and coding fidelity

The supervising SLP reviewed 50% of all recorded intervention sessions to evaluate treatment fidelity and ensure procedures were adjusted to the patient's ability. The supervisor reported that for the sessions she observed/reviewed, there was 100% adherence to the treatment protocol.

Another graduate student clinician reviewed the coding of all written transcripts to ensure the classification of sentence complexity, words per sentence, and error type had a 100% agreement across clinicians.

### 2.9 Data analysis

All writing samples were coded for simple sentences, complex sentences, the mean number of words per sentence, errors, and awkward wording. A count of each variable was calculated for each sample, and an average for each variable and condition was calculated for the first five and the final five sessions. To report the number of simple sentences, complex sentences, and errors, the percentage of errors was calculated for each session and condition. The average number of words per sentence was calculated by dividing the total number of words produced by the total number of sentences separately for each session and condition.

Non-statistical comparisons of the written output were compared between conditions. Additional comparisons were made between output generated during the initial five sessions compared to the final five sessions (for each condition), providing additional information about changes with treatment.

Responses to the confidence and attitude surveys were aggregated. Likert scale responses were entered into a table, with averages calculated for the first and second halves of the intervention. Although a thematic analysis was planned for the open-ended responses, the patient provided short, vague answers (e.g., “it's fun”, “I like it”, “very effective”), which were deemed insufficient for analysis. The patient fully answered the Likert questions for all ten sessions, but he only answered the open-ended questions in 4 out of 10 sessions. Instead of the thematic analysis, the patient's responses will be summarized.

## 3 Results

### 3.1 Changes in written output without ChatGPT

**Figure 3:**
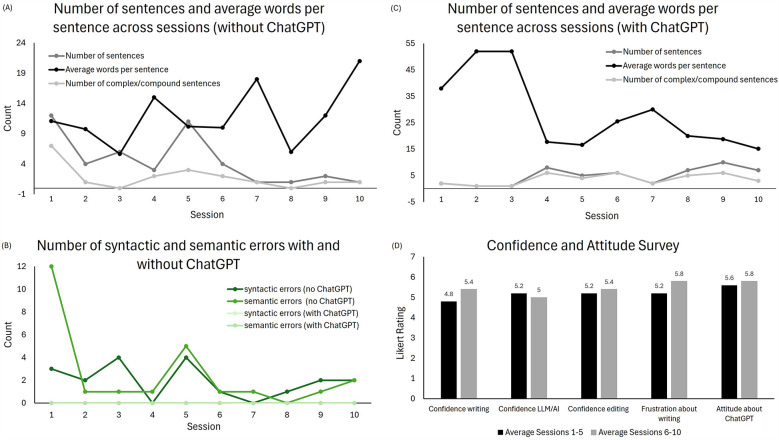
Treatment outcomes: **(A)** number of sentences and mean number of words per sentence produced without ChatGPT, **(B)** syntactic and semantic errors in both treatment conditions across all sessions, **(C)** number of sentences and mean number of words per sentence produced with ChatGPT, and **(D)** patient ratings on the confidence and attitude survey.

The number of sentences produced decreased, while words per sentence increased (Table 1; Figure 3A). Verb errors, pronoun errors, and awkward wording decreased, whereas syntactic and punctuation errors increased. Semantic errors dropped from an initial high of 12 to no more than 0–2 in the final five sessions (Table 1; Figure 3C).

**Table 1:** Treatment (Tx) session data including total count of sentences produced, average for simple, complex/compound sentences produced, words per sentence, and errors for the first five and final five sessions for written output generated with and without ChatGPT.

Measured variable	Without ChatGPT		With ChatGPT	
Sentences produced (Total Count Across Sessions)				
	First 5 Tx sessions	Final 5 Tx sessions	First 5 Tx sessions	Final 5 Tx sessions
All sentence types	36	9	17	32
Sentence data (Average)				
	First 5 Tx sessions	Final 5 Tx sessions	First 5 Tx sessions	Final 5 Tx sessions
Simple sentences	4.6	0.8	0.6	2
Complex & compound sentences	2.6	1.0	2.8	4.4
Average total sentences	7.2	1.8	3.4	6.4
Words per sentence	10	13	35	22
^a^Error data (Count/Total Sentence)				
	First 5 Tx sessions	Final 5 Tx sessions	First 5 Tx sessions	Final 5 Tx sessions
Semantic errors	20/36 (55.6%)	5/9 (55/6%)	0/17 (0%)	0/32 (0%)
Verb errors	6/36 (16.7%)	0/9 (0%)	0/17 (0%)	0/32 (0%)
Non-verb syntactic errors	12/36 (33.3%)	6/9 (66.7%)	0/17 (0%)	0/32 (0%)
Punctuation errors	11/36 (30.6%)	6/9 (66.7%)	0/17 (0%)	0/32 (0%)
Pronoun errors	5/36 (13.9%)	1/9 (11.1%)	0/17 (0%)	0/32 (0%)
Awkward wording	16/36 (44.4%)	2/9 (22.2%)	1/17 (5.9%)	1/32 (3.1%)

^a^ Error data reports the total number of errors and the total number of sentences produced across the first 5 sessions or the final 5 sessions, therefore percentages are shown as number of instances divided by total sentences.

### 3.2 Changes in written output with ChatGPT

Without ChatGPT, the number of sentences produced increased, while words per sentence decreased (Table 1; Figure 3B). With ChatGPT, no errors were observed except for awkward wording, which occurred once in each half of the treatment (Table 1; Figure 3C).

### 3.3 Comparison of written output with and without the ChatGPT

The pattern of change over treatment differed for written output with and without ChatGPT, affecting number of sentences, words per sentence, and errors differently. Specifically, while the number of sentences decreased and words per sentence increased without ChatGPT, the opposite profile was found for the other condition.

### 3.4 Patient responses to confidence and attitude survey

Analysis of the patient's responses to the survey revealed slight increases in writing confidence, editing confidence, attitude about AI, and frustration level. The level of confidence using the AI tool decreased slightly (see Figure 3D).

Responses to four open-ended survey questions revealed that the patient enjoyed using ChatGPT and found it helpful for researching, word finding, combining ideas, providing background information, and recalling historical events. In three out of 10 sessions, the patient provided critical feedback, noting that ChatGPT occasionally generated long, complicated sentences. The patient preferred using the “edit the following” prompt for better results.

### 3.5 Functional outcomes

The patient's personal goal was to write an autobiography. The written output generated during therapy sessions were done with this intention. By the end of the semester, enough text had been generated to create a life story book that was printed for the patient using an online service that allows users to create and print personalized photo products and books. The patient printed multiple copies of his book and proudly gave copies to his friends and family.

### 3.6 Long-term follow-up

One year following the completion of this study, the patient indicated that he continued to use ChatGPT, however, his level of independence or success in using the tool were not evaluated. Specifically, the patient reported that with clinician assistance, he is using ChatGPT creatively to write song lyrics and create images. Since his autobiography project is completed, he has not continued to use ChatGPT to edit his writing, which currently includes emails and social media posts.

## 4 Discussion

### 4.1 Outcomes and implications

The patient showed an increase in the number of total sentences and complex sentences when ChatGPT was used and as treatment progressed. This increase may reflect more comfort using prompts given on the flow chart, including the preferred prompt, “edit the following” and increased confidence using ChatGPT. The use of the AI tool also eliminated semantic, syntactic, punctuation, and pronoun errors.

Without ChatGPT, sentence production decreased while words per sentence increased. Semantic and syntactic errors generally declined, though with some variability. There was also a small increase in the number of complex sentences written. This finding, though intriguing, warrants further examination from a neurorehabilitation perspective. One possible mechanism is cognitive load, as sentence complexity increased, the patient may have been engaging more cognitive resources in the production process, leading to fewer overall sentences but more words per sentence. This increase in complexity might also reflect changes in higher-level linguistic abilities, which can also lead to more grammatical and punctuation errors as the individual works to organize more complex thoughts. The pattern of error reduction in certain areas (e.g., verb and pronoun errors, awkward phrasing) might indicate compensatory strategies emerging as the patient learned to navigate writing challenges without AI support. The small increase in the number of complex sentences and the decline in semantic and syntactic errors, despite the increased difficulty, could reflect improvements in neuroplasticity and cognitive functioning over the course of treatment. These qualitative and quantitative improvements suggest that, while the primary goal was to train the patient to use ChatGPT as a writing aid, there were incidental gains in the patient's linguistic abilities that may have resulted from the cognitive demands and adaptive strategies required during the process.

The patient expressed a consistent enjoyment of using ChatGPT and this aligned with his responses to the attitudes and confidence survey. This patient was very technologically savvy and had prior experience with technology. Previous experience and comfort with technology may be a factor when considering the use of LLMs, such as ChatGPT, to address writing goals.

These results align with prior research on writing challenges in aphasia, particularly in sentence production, complexity, and editing. While computer-based aids support word generation and revision (17), ChatGPT can extend these benefits by improving productivity and reducing errors. In this case, self-written text had fewer but longer sentences, while ChatGPT-assisted writing increased sentence production with shorter sentences. An increase in the length of self-written sentences may reflect improved language abilities. Increased sentences with shorter length using ChatGPT may be due to more effective use of the AI tool/ The patient found ChatGPT helpful for word finding, structuring ideas, and editing, reinforcing its role in lexical retrieval (7, 12). Though syntactic and punctuation errors persisted in self-written text, overall errors decreased, suggesting improved accuracy. The patient's boost in confidence and enjoyment demonstrate ChatGPT's potential to enhance motivation. These findings support LLMs as a valuable tool in aphasia rehabilitation, complementing traditional therapy and promoting independent writing.

The role of the clinician in this case study was substantial, with the patient requiring cues, prompts, and at times even modeling, to effectively use ChatGPT for writing and editing tasks. He relied on structured supports, such as the visual flow chart (See Supplementary Appendix A) and prewritten prompts, to guide his input and generate content, which limited his independence. One year after completing the study, the patient reported continued use of ChatGPT with clinician assistance for creative tasks like writing song lyrics and creating images, but his ability to use the tool independently was not noted. He also did not continue using ChatGPT for editing his writing emails and social media posts. These findings highlight how clinician support may influence both the patient's autonomy and the generalizability of AI tools like ChatGPT for independent use. While AI tools can be beneficial in structured settings, future studies should explore the impact of varying levels of support on long-term outcomes including continued use of AI tools and broader application in therapeutic contexts.

Framing the findings within the ICF model highlights the importance of evaluating interventions like ChatGPT beyond impairment-level outcomes. While changes in sentence production, complexity, and error patterns suggest some shifts in linguistic performance, the patient's engagement with ChatGPT also supported participation in the patient's desire to write his autobiography. Long-term, the patient used ChatGPT to engage in additional activities such as songwriting, and image generation. These outcomes reflect improvements in the activity and participation domains of the ICF, even in the presence of ongoing linguistic support needs. Also consistent with the ICF are the specific activities selected which align with the patient's personal preferences, making this approach patient-centered. Moreover, the structured scaffolding provided by the clinician can be viewed as addressing environmental factors, a key component of the ICF model, by helping the patient overcome barriers to expression and communication. This broader perspective underscores the potential of AI tools not just to remediate deficits but to enhance quality of life and communicative participation.

ChatGPT offers a dynamic platform that may assist PWA in achieving writing goals. Although ChatGPT occasionally generated long sentences, prompts like “reduce the word count” or “improve readability” can guide output. Clinicians using AI tools for PWA may consider setting word limits or constraints before writing tasks, such as: “Please edit sentences for length and readability, and use short, simple sentences for the next hour.”

### 4.2 Limitations and future directions

Since the patient was eight years post-stroke with chronic aphasia, the improvement observed is unlikely due to spontaneous recovery. It is unclear whether improvement resulted from participation in a language-focused intervention, the specific intervention methods utilizing ChatGPT, or both. The influence of the patient's prior experience with technology and language skills remains unknown, as does the likelihood of similar responsiveness in other PWA. While language-based interventions, with or without AI, are expected to enhance written output, the extent to which improvements in this case stem from AI vs. traditional intervention is uncertain.

Reported treatment data includes averages, counts, and percentages. Averages can potentially be misleading if the data do not have a normal distribution. To compensate for this potential limitation, the current study reported some results from each session, not only averages across sessions.

The open-ended survey responses were limited in content. Future studies should revise the design of the survey to elicit better qualitative feedback, such as the impact of intervention, including generalization to other writing and language activities or impact on functional communication, the structure of the intervention sessions, the dosage, and the supports and guidance provided by the clinician.

All case studies have inherent limitations. Findings may not generalize to other patients. Here, it is unknown if outcomes would differ for PWA with different communication profiles or linguistic abilities. These current results motivate future research, which should include a larger sample of patients with chronic aphasia and varied profiles of aphasia to determine if writing intervention utilizing LLMs improves written output and if such improvements generalize across writing activities.

The results of this study also motivate future research that modifies the intervention methodology, specifically using a smaller number of LLM prompts, compared to the complex flow chart used in this study. A streamlined flow chart with fewer prompts may be easier for PWA to use and may result in greater independent use of AI tools.

## 5 Conclusion

This case report evaluates the use of ChatGPT to improve written expression in a patient with chronic aphasia, showing improvements in error count, sentence production, and words per sentence. Without ChatGPT, sentence production decreased and words per sentence increased, suggesting greater complexity. With ChatGPT, the patient produced more sentences with fewer words per sentence, indicating increased productivity. Survey results revealed the patient enjoyed using ChatGPT and gained confidence in writing and AI-assisted editing. This structured approach improved both productivity and quality, highlighting AI's potential in aphasia treatment and motivating further research. Clinicians may consider AI tools like ChatGPT to support written communication in PWA, particularly those with prior technological experience.

## Data Availability

The datasets presented in this article are not readily available because of ethical and privacy restrictions. Requests to access the datasets should be directed to the corresponding author.
