# Supplementary figures and images for: Harnessing AI for aphasia: a case report on ChatGPT's role in supporting written expression
Source: Front Rehabil Sci. 2025 May 30;6:1600145. doi: 10.3389/fresc.2025.1600145 (PMC12163031; doi:10.3389/fresc.2025.1600145)

# Appendix A


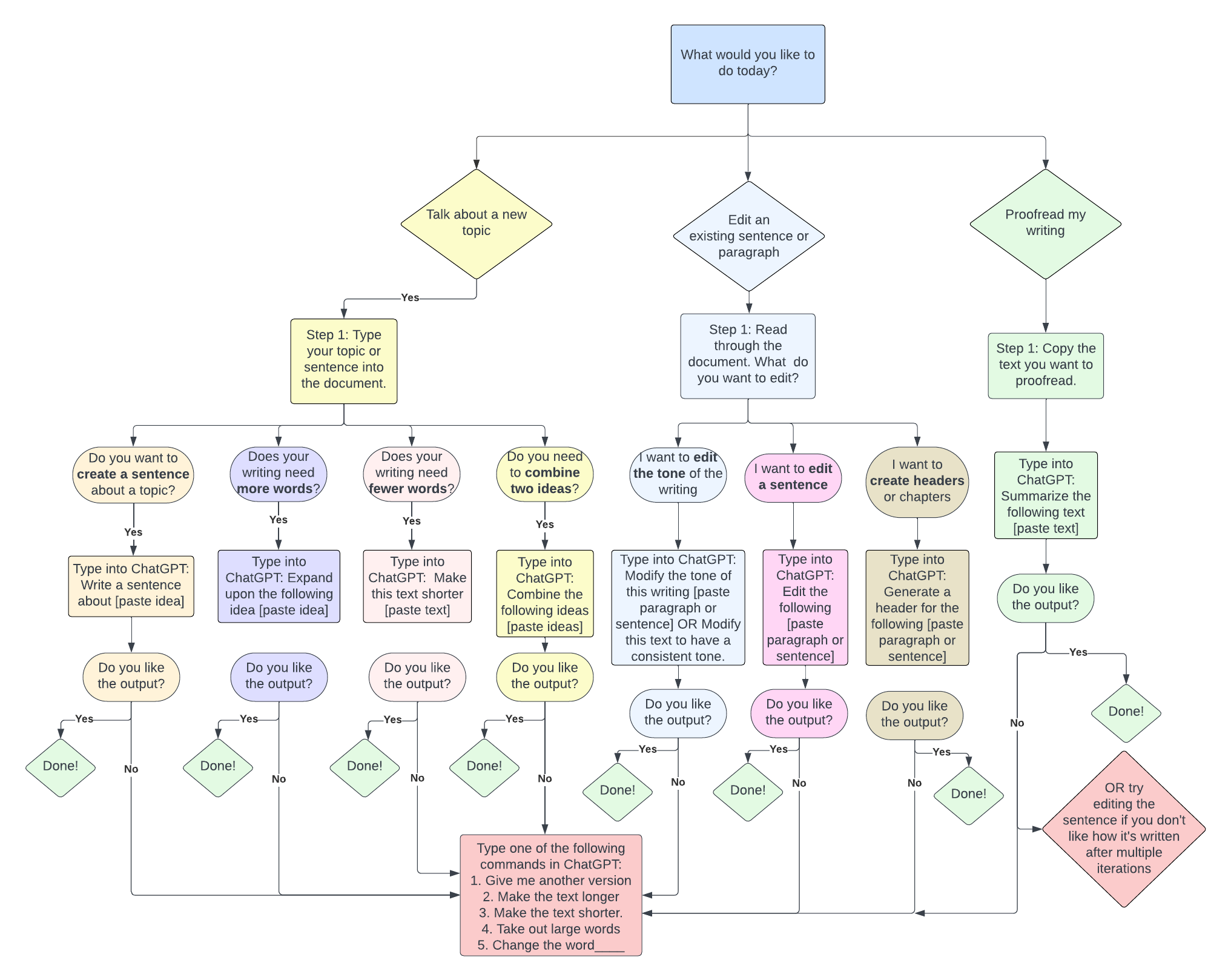


# Appendix B


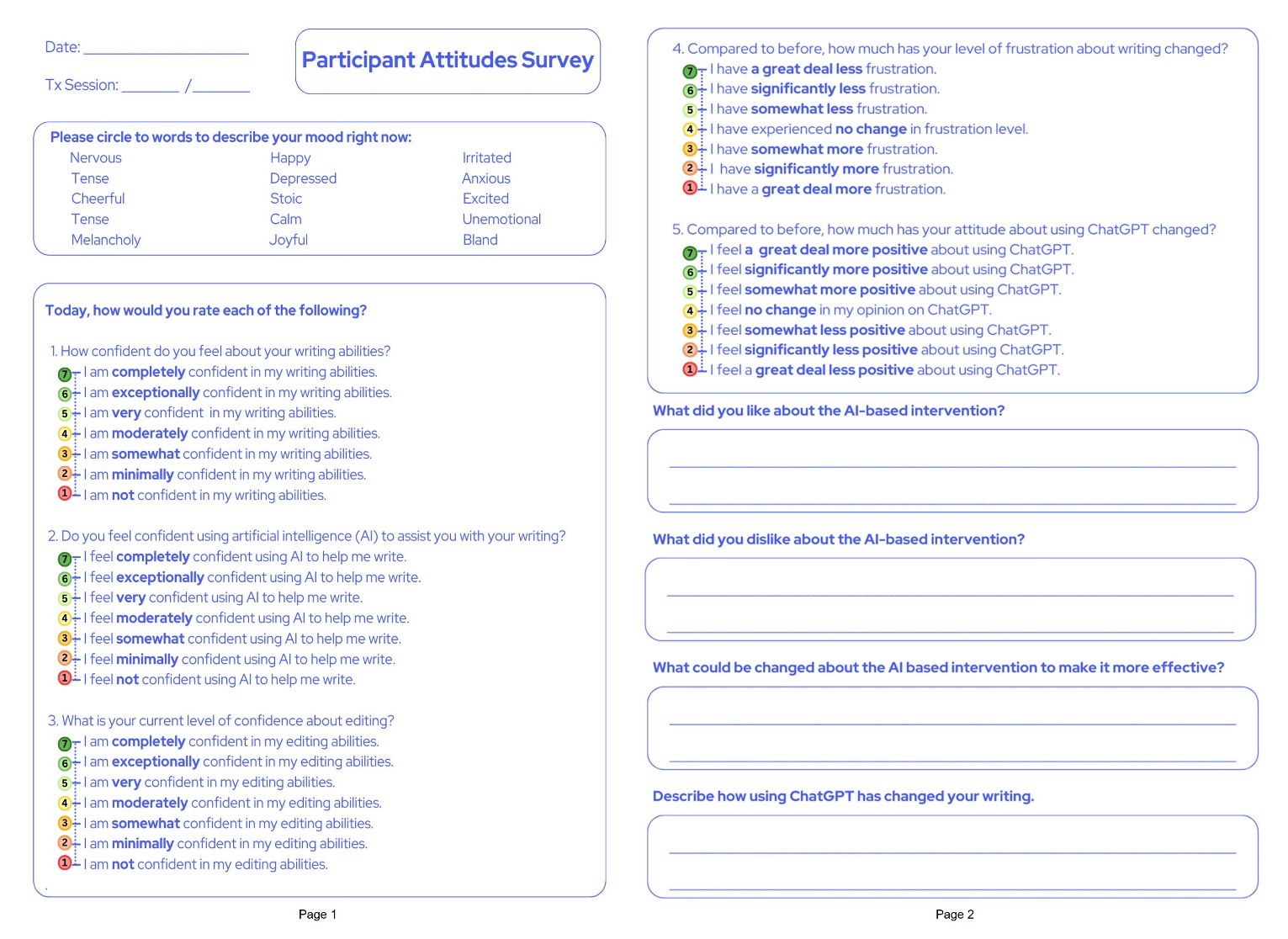

Supplement: Supplementary file 1 [file Supplementaryfile1.docx]
